# Revealing cancer initiating cells in metastatic melanomas by harnessing the host′s anti tumor humoral immune mechanisms

**DOI:** 10.1186/1479-5876-12-S1-O1

**Published:** 2014-05-06

**Authors:** Beatrix Kotlan, Gabriella Liszkay, Gyorgy Naszados, Judit Olasz, Szabolcs Horvath, Klara Eles, Miri Blank, Yehuda Shoenfeld, Laszlo Gobor, Laszlo Toth, Orsolya Csuka, Maria Godeny, Miklos Kasler, Francesco M Marincola

**Affiliations:** 1 Molecular Immunology and Toxicology, National Institute of Oncology, Budapest, Hungary; 2 Oncodermatology, National Institute of Oncology, Budapest, Hungary; 3 Radiological Diagnostics, National Institute of Oncology, Budapest, Hungary; 4 Pathogenetics, National Institute of Oncology, Budapest, Hungary; 5 Surgical and Molecular Tumorpathology, National Institute of Oncology, Budapest, Hungary; 6 Zabludowitz Center for Autoimmune Diseases, Sheba Medical Center affiliated to Sackler Faculty of Medicine, Tel-Aviv University, Tel Aviv, Israel; 7 Oncosurgery, National Institute of Oncology, Budapest, Hungary; 8 Board of Directors, National Institute of Oncology, Budapest, Hungary; 9 SIDRA Medical and Research Center, Doha, Qatar

## Background

To determine whether the growth of tumors is sustained by the whole cancer cell population or it is maintained only by small fraction of cancer initiating cells, has crucial impact on the design of suitable therapies. That is why the research on defining the subpopulation of cancer initiating cancer stem cells (CSC) in solid tumors has come into highlights.

## Materials and methods

A complex panel assay at cellular and molecular levels has been performed on primary and metastatic cancerous tissue biopsies and peripheral blood of patients with malignant melanomas (n = 153) (Ethycal permission: ETT TUKEB 16462- 02/2010).

## Results

Cell cultures grew out of the great majority of the starter metastatic tissue specimen and cancer initiating cells could be sorted (BD FACSAvia Sorter) by colocalized unique sialilated glycosphingolipids and anti CD20 binding capacity. Characteristic growth pattern, spheroid forming, CSC markers like CD133, Nestin, ABCB5, CD20 and unique GD3 gangliosides were found. Patients′ sera and selected patients′ Epstein Barr Virus transformed cell supernatants in bulk or after limiting dilution cloning were tested for cancer binding by ELISA and immunofluorescence. Our novel tumor infiltrating B cell antibody phage display technique and DNA sequence cluster analysis (Vector NTI Advance 11, Bioedit 7.0, ClustalX2.0.11) resulted in some antibody fragments, belonging to representative tumor binding antibody variable region subgroups, with defined silalilated glycosphingolipid specificity.

## Conclusion

Our strategy enables the detection and characterization of cancer stem cells in metastatic melanomas, with potential diagnostic importance. The novel peripheral blood and tumor infiltrating B cell antibody profile analysis proved to be a useful asset to reveal anti tumor humoral immune responsiveness and harness it by antibody engineering technique for further diagnostic and therapeutic usage.

